# Lowback Pain Management with a Combination of Uridine Triphosphate, Cytidine Monophosphate, and Hydroxocobalamin: A Systematic Review and Meta-Analysis

**DOI:** 10.1055/s-0045-1804495

**Published:** 2025-07-10

**Authors:** Marco Antonio N. Mibielli, Mendel Suchmacher, Mauro Geller, Spyros G. E. Mezitis, Carlos P. Nunes, Aline Sintoveter

**Affiliations:** 1 Centro Universitário Serra dos Órgãos, Teresópolis, RJ, Brazil; 2 Postgraduate Program in Immunology, Instituto de Pós-graduação Médica Carlos Chagas, Rio de Janeiro, RJ, Brazil; 3 Endocrinology and Internal Medicine, Cornell University Medical School, New York, NY, United States; 4 University of Central Florida College of Medicine, Orlando, FL, United States

**Keywords:** cytidine monophosphate, hydroxocobalamin, low back pain, spinal nerve roots, uridine triphosphate, dor lombar, hidroxocobalamina, monofosfato de citidina, raízes nervosas espinhais, uridina trifosfato

## Abstract

Low back pain is a common complaint. This syndrome comprehends different underlying mechanisms, which are difficult to differentiate in a timely manner only through semiotic, laboratory, and imaging resources available in an emergency setting. Such circumstances make practitioners prone to an initial symptomatic approach in the form of medications (non-steroid anti-inflammatory drugs, analgesics, muscle relaxants) or local procedures (local heat, massage). Peripheral neurotrophic substances, such as pyrimidine nucleotides (uridine triphosphate and cytidine monophosphate) combined with vitamin B12 (hydroxocobalamin), have been used as anabolic precursors able to provide spinal nerve roots with triggering elements useful for nerve and glial cells regeneration, once a likely spinal compression mechanism is contained. The authors performed a systematic review and meta-analysis with the above combination with the aim of better determining its role in low back pain management.

## Resumo

A dor lombar é uma queixa comum. Essa síndrome tem diferentes mecanismos subjacentes, difíceis de diferenciar em tempo hábil apenas por meio dos recursos semióticos, laboratoriais e de imagem disponíveis no atendimento de emergência. Isso faz com que os profissionais tendam a uma abordagem sintomática inicial composta por medicamentos (anti-inflamatórios não esteroidais, analgésicos, relaxantes musculares) ou procedimentos locais (calor local, massagem). Substâncias neurotróficas periféricas, como nucleotídeos de pirimidina (trifosfato de uridina e monofosfato de citidina) combinados com vitamina B12 (hidroxicobalamina), têm sido usadas como precursores anabólicos capazes de fornecer às raízes nervosas espinhais elementos desencadeadores úteis para a regeneração de neurônios e células da glia, uma vez que um provável mecanismo de compressão espinhal seja contido. Os autores realizaram uma revisão sistemática e meta-análise com a combinação acima com o objetivo de determinar melhor seu papel no tratamento da dor lombar.

## Introduction

Low back pain (lumbosacral pain) associated with compression neuropathy syndromes represents one of the most frequent pathological manifestations of the spine. Low back pain is a rather unspecific term that may comprehend distinct entities clinically expressed in an isolated, combined, or overlapping fashion. 1 Its prevalence ranges from 30 to 70% among the 18-to-74 year old population. 2 Sciatica, on its turn, is a general term used to refer to lumbar radicular pain, often radiating unilaterally to the leg according to the corresponding dermatome. It can be accompanied by motor, sensitive, and/or reflex deficits. Pain is worse than “classic” low back pain, and the chronicity risk is higher. 3 Several therapeutic modalities—conservative, pharmacological, and invasive—have been developed and applied over the last 100 years. The uridine triphosphate (UTP), cytidine monophosphate (CMP), and hydroxocobalamin combination has been prescribed in some countries for the symptomatic control of this syndrome in the last 50 years. The objective of the present systematic review and meta-analysis is to measure the effects of the combination in this setting.

## Materials and Methods

### Primary Studies Search and Selection

**Fig. 1:**
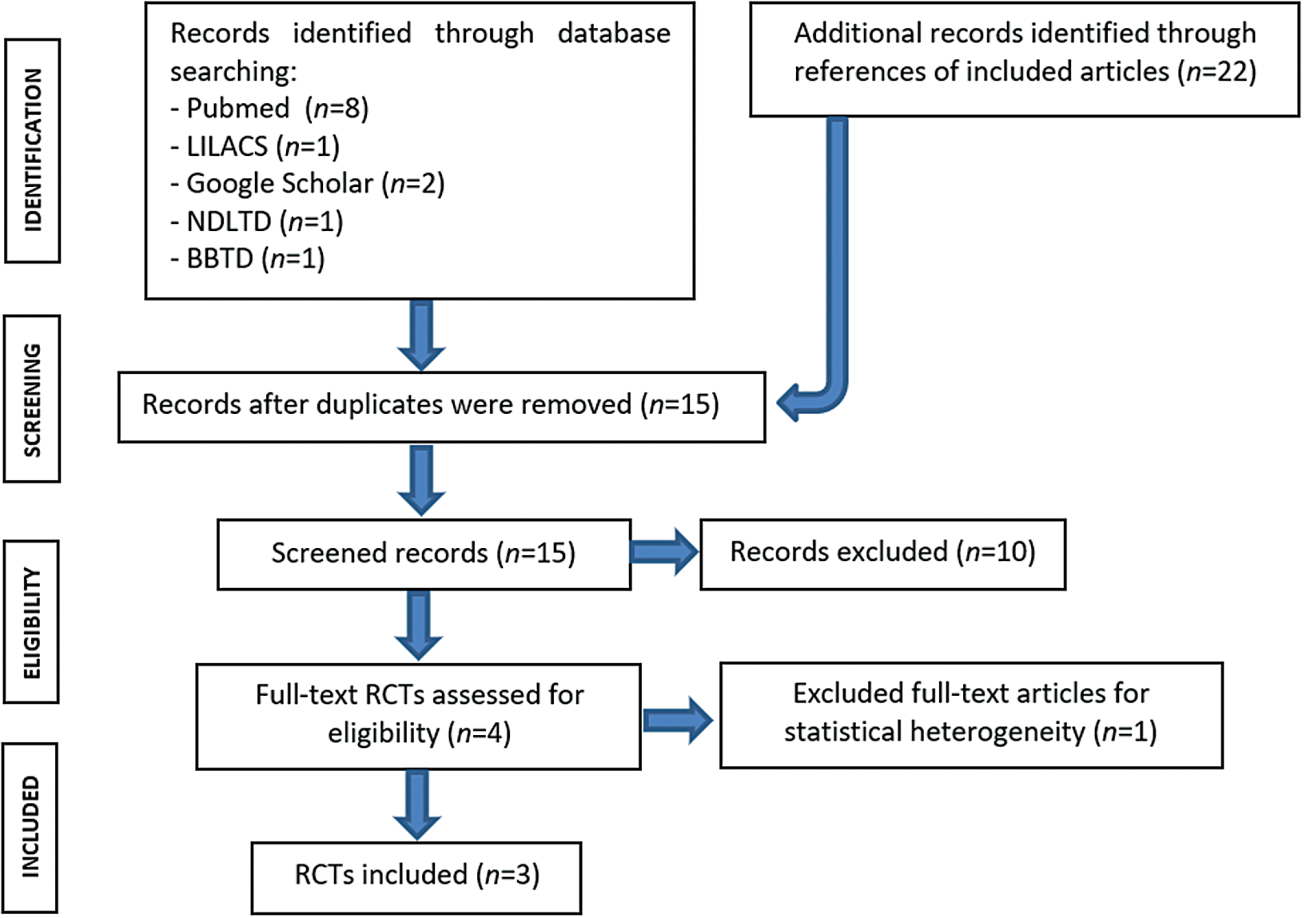
Diagram for study selection as applied in the current systematic review and meta-analysis.

The study was performed by two independent researchers who worked in parallel and blindly, both according to the following parameters: (1) epidemiological studies, observational studies, randomized clinical trials (RCTs), non-RCTs, systematic reviews, and meta-analyses as study types; (2) no language or year of publication restrictions; and (3) the names of the authors of the primary studies were not regarded (even though personal consulting was permissible). Supporting literature, such as textbooks, basic scientific papers, and pharmacological compendiums, was consulted when deemed necessary (not accounted for systematic review purposes). The studies search was performed according to the guidelines of the Preferred Reporting Items for Systematic Reviews and Meta-analyses (PRISMA) statement. 4 Flowchart is depicted in Fig. 1 (details on the scrutinized sources are listed in the Appendix ).

**Appendix 1:** Sources analyzed and their respective parameters

1. Pubmed: (1) uridine triphosphate, UTP, cytidine monophosphate, CMP, hydroxocobalamin, vitamin B12, spinal nerve root, and pyrimidine nucleotides in the title, and/or (2) low back pain, lumbago, lumbopelvic pain, or sciatica anywhere in the text; 2. LILACS: (1) *uridina trifosfato* , UTP, *citidina monofosfato* , CMP, *hidroxocobalamina* , *vitamina B12* , *raiz dorsal* , *nucleotídeos pirimidínicos* in the title, and (2) *lombalgia* , *dor lombar* or *ciatalgia* anywhere in the text; 3. Google Scholar: (1) uridine triphosphate, UTP, cytidine monophosphate, CMP, hydroxocobalamin, vitamin B12, spinal nerve root, and pyrimidine nucleotides in the title, and or (2) low back pain, lumbago, lumbopelvic pain, or sciatica anywhere in the text, and (3) up to three search pages; 4. NDLTD: uridine triphosphate, UTP, cytidine monophosphate, CMP, hydroxocobalamin, vitamin B12, spinal nerve root, and pyrimidine nucleotides in the title; 5. BBTD: *uridina trifosfato* , UTP, *citidina monofosfato* , CMP, *hidroxocobalamina* , *vitamina B12* , *raiz dorsal* , or *nucleotídeos pirimidínicos* in the title; 6. Bibliographic references from the selected publications.

### Endpoints and Outcomes Collection

The researchers' results were crossed by a reviewer for validation, who reported no conflicts between the body of findings of the former two. The studies were selected according to their respective titles and abstracts, as per the following parameters of interest: (1) low back pain, (2) UTP, CMP, and hydroxocobalamin combination in its symptomatic control, (3) efficacy and safety comparison with vitamin B12 (hydroxocobalamin and cyanocobalamin), and (4) additive and/or synergistic pharmacological properties of UTP, CMP, and hydroxocobalamin combination. Text search was extended from the title/abstract to the body of the text when searchers felt necessary. No personal contact with the studies' authors was necessary. A comprehensive literature on the general pharmacology of UTP, CMP, and hydroxocobalamin was also retrieved.

We followed the Preferred Reporting Items for Systematic Reviews and Meta-Analyses (PRISMA) guidelines to perform the present study. 4 The Visual Analog Scale (VAS) measurements were chosen as study endpoint. The mean difference between the baseline and final VAS, along with standard deviation were used for the UTP, CMP, and vitamin B12 group, as well as the vitamin B12 monotherapy group, with 95% confidence intervals (CIs). The Cochran Q test and I2 statistics were used to assess heterogeneity. *P* -values < 0.10 and I2 statistics ≥ 50% were considered to determine the significance of heterogeneity, and the use of a random-effect model. One author conducted the statistical analyses using R software (R Foundation for Statistical Computing, Vienna, Austria). 5

## Spinal Nerve Root Injuries and Chronic Low back Pain

### Definition

Low back pain can be defined as a midline pain spanning from the lowest rib down to the gluteal fold. This syndrome can be classified as acute (< 6 weeks), subacute (6–12 weeks), or chronic (> 12 weeks duration). It can be accompanied by mobility loss, radiation to the legs, groin, and posterior pelvis, mood change, and disturbances in social interactions. 1 2

### Low back Pain Pathophysiology

Low back pain is the outcome of a pathophysiologic complex process involving neural (spinal nerve roots and dorsal root ganglia) as well as somatic (intervertebral facet joints, periosteum, ligaments, tendons, fasciae, paravertebral muscles, and intervertebral discs) structures 6 . In most cases, both mechanisms overlap. Even though a culprit structure cannot be pointed out in 80 to 90% of cases, in 10 to 15% of patients, neural tissue involvement can be demonstrated. 1 2 Herniated discs, chronic spinal degenerative conditions, and spinal stenosis are the most common etiological mechanisms found in clinical practice. 1 6 Acute mechanical compression exerted by a vertebral pedicle on a lumbosacral spinal nerve root and/or dorsal root ganglion can lead to local vascular compromise, microstructural changes, and inflammation, all linked to sensory deficits, pain threshold decrease, as well as loss of somatic strength and autonomic (bladder and bowel) control in the corresponding dermatome. 7 Pain onset is proportional to the degree of extrinsic compression and spinal nerve root irritation, both difficult to determine in the clinical setting. 6 8 During convalescence, local inflammatory cytokines and neurotrophic factors released for neural tissue healing can decrease pain threshold by promoting neuroplasticity and stimulating uninjured neighboring neurons, with paradoxical algic worsening. 8

### Clinical Picture, Diagnosis and Prognosis

If present, neuropathic pain manifests itself as paresthesia, hyperesthesia, allodynia, and hyperalgesia. Pinpointing the pain structural origin can be challenging since pathophysiological mechanisms overlap and evolve dynamically. Similarly, maneuvers such as digital compression and mobility tests are limited due to semiotic inaccessibility of the structures potentially involved as well as poor discriminatory power. 1 2 Differential diagnoses with low back pain are local fracture (trauma, fall from height, preexisting osteoporosis), infections (B symptoms, immune suppression, IV drug abuse), or tumoral disease (B symptoms, pain that increases in supine position, paraproteinemia). 2 Imaging is generally unnecessary in the early stages of low back pain presentation. Nevertheless, if indicated, the assisting physician should take into consideration that segmental and muscle dysfunction, as well as sacroiliac joint syndrome, are not amenable to morphologic demonstration. 2 6 Low back pain resolves within 4 to 6 weeks in 50% of cases and in 12 weeks in 80% of cases. On the other hand, recurrence and inability to work are common features if the etiology is not approached. 2

### Neuroregeneration and Lumbosacral Pain

Dorsal root ganglia have axons that reach laterally and medially towards the spinal nerve roots and spinal cord, respectively. Injuries to the former induce a regenerative process, similar to compressive neuropathic lesions while injuries to the latter do not result in regeneration due to the inhibiting environment of the central nervous system (CNS). 6 Presuming that spinal nerve roots' axons present a biology similar to that of peripheral nerves, one can assume that both might share similar regenerative patterns. Therefore, a combined Seddon and Sunderland classification of peripheral nerve injuries could be proposed for classifying spinal nerve root injuries in the context of low back pain ( Table 1 ).

**Table 1:** Combined Seddon and Sunderland classifications of peripheral nerve injuries 9

Sunderland	Seddon	Pathophysiology	Recovery
First degree	Neuropraxia	Segmental demyelination	Full
Second degree	Axonotmesis	Axon severed, intact endoneurium	Full
Third degree	−	Axon severed, compromised endoneurium, intact perineurium	Variable
Fourth degree	−	Axon, endoneurium, perineurium discontinuity, intact epineurium	Null
Fifth degree	Neurotmesis	Discontinuity of entire nerve	Null
Sixth degree	−	Mixed	Unpredictable

### Therapy and Prognosis

Conservative measures include physical therapy (spinal traction, stretching, massage), relaxation techniques, cognitive behavioral therapy, transcutaneous electrical nerve stimulation (TENS), exercising, bedrest, or simply resuming normal daily activities. Preventing muscular contractures is paramount as these can delay function recovery. Medical treatment for the neuralgic component of the syndrome includes antidepressants (gabapentin, oxcarbazepine, and lamotrigine), and pregabalin. Somatic pain component can be treated with paracetamol or non-steroid anti-inflammatory drugs (ibuprofen, diclofenac, naproxen). 1 2 6 Healing from mechanical injury against a spinal nerve root depends on the specific structure involved, the degree of the insult, its mechanism and duration. Even with optimal management, recovery is typically incomplete and dysfunctional, and neuropathic pain can persist. Currently, there are no recognizable prognostic factors for motor recovery or pain resolution associated to these types of lesion. 8 9 10

## Pyrimidine Nucleotides – UTP and CMP

**Fig. 2:**
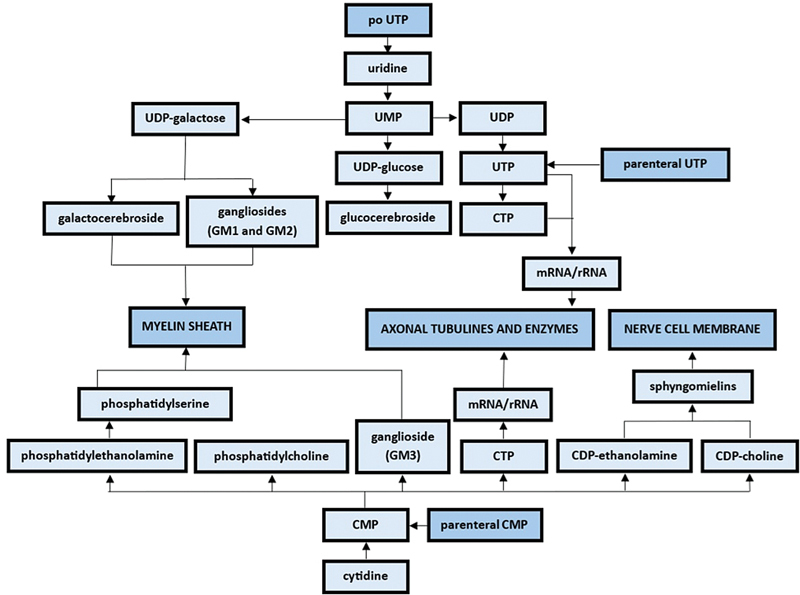
Nerve cells and Schwann cells biochemical pathways amenable to pharmacological influence from pyrimidine nucleotides.

A nucleotide structural model has been used in the development of several pharmacologically active substances, such as antitumoral metabolites (e.g., mercaptopurine), nucleoside analogs (e.g., lamivudine), and nucleoside antiarrhythmics (e.g., adenosine). In the context of compression neuropathies, pyrimidine nucleotides UTP and CMP are used as metabolic neurotrophic substances involved in the synthesis stimulation of nerve cell membrane, myelin sheath, and axonal proteins (tubulines and enzymes), as demonstrated in Fig. 2 .

### Pyrimidine Nucleotides Pharmacodynamics

**Increase in nerve cells protein synthesis.** Wallerian degeneration is expected to follow the disintegration of axons and Schwann cells after a mechanical trauma on a peripheral nerve or nerve root cell. The velocity of soma and myelinic anabolic pathways is correspondingly accelerated during this phenomenon. Therefore, the quantity of nucleotides consumption is expected to be greater in comparison to nerve cells and glial cells in their steady state, along with other vital metabolites. 11 12 13 14

**Increase in myelin sheath synthesis.** In the later stages of Wallerian degeneration, the passage of axonal regeneration cones through the Bungner band at the distal neural stump will trigger the wrapping of Schwann cells membrane around the advancing axons, then forming a new myelin sheath. The demand for myelin lipids precursors in Schwann cells, among them nucleotide intermediary metabolites, is expected to be increased under the above-described conditions. 13 15 16

**Increase of nerve cell membrane synthesis.** Similarly to the mechanism of myelin sheath increase synthesis, an increase in axolemmal structural elements is supposed to take place, in parallel to regenerative cones' progression. Integration of *de novo* nucleotide metabolic intermediaries in this scenario will be necessary as well. 14 16 17 18 19 20 21 22 23

### Pyrimidine Nucleotides Safety

Uridine triphosphate and CMP are contraindicated in the acute phase of ischemic stroke due to the possibility of nerve-cell membrane phosphatidylcholine degradation into diacylglycerol and free fatty acids, under brain anoxia conditions. 24 25 Individuals with dihydropyrimidine dehydrogenase or ornithine carbamoyl transferase deficiencies may present excessive pyrimidine nucleotides in the CNS. Therefore, UTP and CMP are contraindicated in patients who present the above-described conditions. 16

## Hydroxocobalamin

Hydroxocobalamin is a manufactured injectable form of vitamin B12. It is involved in the so-called one-carbon metabolism reactions (cystein, methionine, and pyrimidine nucleotides synthesis, as well as methylation reactions), mitochondrial metabolism, and myelin basic protein synthesis (myelin sheath structural stabilization). Vitamin B12 analgesic properties are still a matter of debate, but, seemingly, it does speed up low back pain improvement due to its participation in myelin sheath recovery. 26 Hydroxocobalamin's adverse reactions are acneiform erythema, fever, exanthematous hot flashes, blood hypertension (intravenous injections), peripheral edema, photosensitivity, pruritus, and hives (case reports). 27 28 29

Spinal nerve root compression syndromes present a complex pathophysiology, hinting potential targets for different therapeutic modalities. As shown in Fig. 2 , UTP and CMP share synergistic effects over regenerating peripheral nerve metabolic pathways, whose myelin sheath synthesis arm can be additively influenced by hydroxocobalamin as it promotes MPB synthesis. Assuming that neuropathic low back pain can also be triggered by the disintegration of the microstructures of nerve and Schwann cells of the spinal nerve root, one can presume that their anticipated resynthesis could provide a sooner pain improvement.

## Results

We retrieved a total of 30 general studies and 5 clinical trials on UTP, CMP, and hydroxocobalamin (4 RCT and 1 non-RCT) in low back pain management, the latter ones comprehending a total of 1,236 patients (no epidemiological studies, observational studies, systematic reviews, or meta-analyses were found). Reported research endpoints were: (1) Visual Analog Scale (VAS) (selected endpoint for meta-analysis as a 0–100 mm visual scale), (2) Patient Functionality Questionnaire (PFQ), (3) percentage of patients presenting improvement on PFQ, (4) percentage of patients presenting improvement on VAS, (5) patient global evaluation, (6) physician global evaluation, (7) Roland-Morris Questionnaire, and (8) finger-to-floor distance. In 3 RCTs, the combination was found effective in reducing VAS versus a comparative; 30 31 32 in 1 RCT, it was found to be less effective versus the same combination comprising diclofenac-cholestyramine, 26 and in 1 trial, it was found to be effective in a self-paired design. 32 In all 5 trials, the combination was declared as safe. The findings related to the above-mentioned studies are summarized in Table 2 .

**Table 2:** Selected RCTs, non-RCTs, and self-paired study on the combination of UTP, CMP and hydroxocobalamin* for the management of spinal nerve compression syndromes

Authors	Study objectives	Regimens	Study type	*n*	Results	Safety	Conclusion
** Goldberg et al. 29 (2009) **	To assess a combination of UTP, CMP, and hydroxocobalamin in the treatment of neuralgia due to degenerative orthopedic spine alterations with neural compression	Group A: 2 capsules of UTP, CMP, and hydroxocobalam. Group B: 2 capsules of hydroxocobalamin 1,000 mcg. Both regimens tid for 30 days	Double-blind and randomized	*n*_A _ = 40 *n*_B _ = 40	There was VAS reduction in both groups, though significantly greater in group A ( *p* < 0.0001)	Adverse events were considered mild to moderate, with a statistically better overall performance with group B	Group A combination presented a positive effect in the parameter of pain on degenerative orthopedic spine alterations with neural compression
** Mibielli et al. 26 (2010) **	To evaluate the efficacy and safety of UTP, CMP, and hydroxocobalamin in the treatment of acute, non-traumatic low back, hips, and neck pain	Group A: (1) box A containing 6 capsules of UTP, CMP, and hydroxocobalamin, and (2) box B containing 2 capsules of diclofenac-cholestyramine. Group B: (1) box A containing 6 capsules of UTP, CMP, and hydroxocobalamin, and (2) box B containing 2 capsules of placebo. Box A taken as 2 capsules tid, and box B as one capsule bid, for both groups, for 10 days	Double-blind and randomized	*n*_A _ = 40 *n*_B _ = 41	Group A combination resulted in a higher number of subjects with VAS score reduction > 30 mm in comparison with group B ( *p* < 0.0006)	The number of subjects presenting adverse events did not vary significantly between groups	Group A combination reduced pain among subjects with non-traumatic low back, hips, and neck pain
** Mibielli et al. 32 (2014) **	To corroborate analgesic effects of UTP, CMP, and hydroxocobalamin observed in group B of Mibielli et al. 26 (2010) trial	Box A containing 6 capsules of UTP, CMP, and hydroxocobalamin taken as 2 capsules tid. Box B containing 2 capsules of placebo taken as one capsule bid for 10 days	Self-paired	*N* = 41	The difference between V3 (10 ^th^ therapy day) and pretreatment VAS score was statistically significant ( *p* < 0.0001)	The same as for Mibielli et al. (2010)	Group B combination seems to have analgesic properties in medium-term use in acute, non-traumatic low back, hips, and neck pain
** Goldberg et al. 30 (2017) **	To assess the safety and efficacy of the combination UTP, CMP, and hydroxocobalamin in patients with neuralgia due to degenerative orthopedic alterations and trauma (low back, hip, and carpal tunnel syndrome) associated to neural compression	Group A: 2 capsules of UTP, CMP, and hydroxocobalam. Group B: 2 capsules of hydroxocobalamin 1,000 mcg. Both regimens tid for 30 days	Double-blind and randomized	*n*_A _ = 200 *n*_B _ = 200	There was a statistically significant superiority of group A regimen in VAS reduction ( *p* = 0.0003)	There were transitory adverse events and no severe adverse event in both Groups	Group A combination was safe and effective in the treatment of neuralgias due to degenerative orthopedic alterations associated with neural compression
** Mibielli et al. 31 (2020) **	To compare the efficacy and tolerability of the combination of UTP, CMP, and hydroxocobalamin compared to the combination of thiamine, pyridoxine and cyanocobalamin in patients with low back pain	Group A: 2 capsules of UTP, CMP, and hydroxocobalam. Group B: 2 capsules of thiamine, pyridoxine and cyanocobalamin. Both regimens tid for 60 days	Double-blind and randomized	*n*_A _ = 317 *n*_B _ = 317	VAS score reduction was statistically significant in both Groups at D30 and D60 ( *p* < 0.0001), with a comparative better performance of Group A combination at D30 ( *p* < 0.001)	75 (24%) and 105 (33%) of subjects presented adverse effects in groups A and B, respectively	VAS score reduction was documented both groups' combinations at D30 and D60, with a comparative better performance for group A combination at D30

**Note:** *The capsules of the combination contained 1.5 mg, 2.5 mg, and 1 **,** 000 mcg of UTP, CMP, and hydroxocobalamin, respectively.

**Fig. 3:**
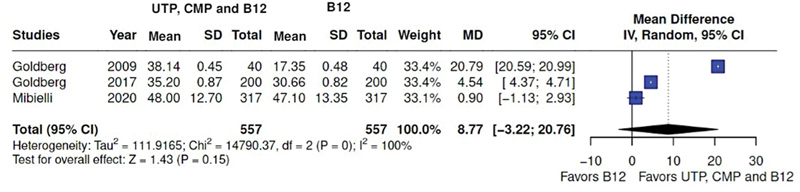
Meta-analysis on UTP, CMP, and hydroxocobalamin combination on low back pain control. Forest plot graph favors the combination over vitamin B12.

Of the 5 trials on the UTP, CMP, and hydroxocobalamin combination detailed in Table 2 , 3 presented comparable outcomes for meta-analysis (mean differences of VAS scale for the combination versus hydroxocobalamin) (median = 8.77; 95%CI: -3.22–20.76). Pooled analysis of the primary studies and corresponding forest plot representation are presented in Fig. 3 .

## Discussion

Low back pain syndromes are frequently presented as chronic conditions that compromise patients' wellbeing, personal productivity, and quality of life. Their complex pathophysiology makes a multi-target therapeutic approach feasible, with an association of drug combinations as a plausible modality. The combination of UTP, CMP, and hydroxocobalamin showed evidence of its efficacy and safety on low back pain control, possibly involving spinal nerve root compression, through several RCTs and the current systematic review and meta-analysis. Their combination in this setting is based on a pathophysiological rationale, expressed through additive and synergistic effects. One limitation of our study was the limited number of RCTs amenable to meta-analysis. Notwithstanding, we consider that our findings warrant the UTP, CMP, and hydroxocobalamin combination as a potentially useful resource for the management of low back pain associated with spinal nerve root compression.

## Conclusion

Based on the results of the current systematic review and meta-analysis, we consider the combination of UTP, CMP and hydroxocobalamin for the management of low back pain associated with spinal nerve root compression to be safe and effective.
